# Editorial: Improving body composition and functional capacity in chronic kidney disease patients

**DOI:** 10.3389/fnut.2023.1223975

**Published:** 2023-07-28

**Authors:** Natalia Tomborelli Bellafronte, Guillermina Barril, Erick P. de Oliveira

**Affiliations:** ^1^School of Dietetics and Human Nutrition, McGill University, Montreal, QC, Canada; ^2^Department of Nephrology, Hospital Universitario de la Princesa, Madrid, Spain; ^3^School of Medicine, Federal University of Uberlandia, Uberlandia, Brazil

**Keywords:** body composition, chronic kidney disease, obesity, protein energy wasting, sarcopenia, exercise, nutrition, physical function

Chronic kidney disease (CKD) is a major global public health problem, with an annual economic burden of more than $114 billion (1). CKD moved from the 21st (1990) to the 11th (2016) position in the world ranking of causes of death and years of life lost (2).

Metabolic disorders present in chronic kidney disease (CKD) lead to increased protein catabolism (3) resulting in loss of muscle mass and function (4). Protein catabolism is worsened by other typical conditions, such as uremia-related gastrointestinal symptoms, physical inactivity, nutrient malabsorption, and nutrient loss into the dialysate (5). Therefore, muscle impairment leading to sarcopenia (6) and protein energy wasting (PEW) is common among patients with CKD (7) and is associated with higher mortality rates (8), decreased physical function and physical activity (9), and worse outcomes (10). Obesity is also common among CKD patients (7) and is associated with higher risk of mortality (11) and disease progression (12).

This recent Research Topic on “*Improving body composition and functional capacity in chronic kidney disease patients*” represents a collection of five original research articles in the field of CKD, ranging from development of more feasible methods to diagnose PEW and central obesity to dietary and exercise interventions aimed to prevent muscle depletion and increase responsiveness to pharmacological treatment, as well as evaluation of the association between obesity and cardiovascular disease (CVD) risk. A common theme across all articles is the importance of body composition and/or nutritional status and its association with clinical outcomes in patients with kidney disease. This is an important Research Topic for investigation since PEW, sarcopenia, and obesity have high prevalence rates among patients with CKD and are associated with adverse clinical outcomes.

Among patients under hemodialysis, 60% are alive 3 years after starting treatment (13). Cardiovascular disease (CVD) is the main cause of death in this population (14), but the nutritional status deterioration (15) as a result of PEW syndrome is also associated with increased death risk from CVD (16, 17). PEW, a modifiable risk factor, is present in 28–54% of patients under dialysis (18). PEW diagnosis is recommended to be done by the International Society of Renal Nutrition and Metabolism (ISRNM) according to assessment of biochemical criteria: low body weight, reduced total body fat, or weight loss; a decrease in muscle mass; and low protein or energy intake (19). However, given the need of meeting multiple criteria, in particular the difficult and usually unreliable estimating of dietary intake (20), the ISRNM diagnosis is rarely implemented in clinical practice. In order to make the diagnosis more objective, Chen et al. analyzed the independent influencing factors of PEW among 380 hemodialysis patients. The three model for prediction of PEW proposed by Chen et al. had an area under the curve from 0.85 to 0.91, and no significant difference was found compared to ISRNM diagnostic criteria (*p* > 0.05) was observed. The novel PEW prediction model is more convenient than the traditional diagnostic criteria and can be applied to identify PEW in HD patients.

Patients under HD treatment suffer from worse muscle function (21), anabolic resistance (22), and poorer protein and calorie balance (23) than patients under other CKD treatments. As a result, sarcopenia is more prevalent among patients undergoing dialysis (30%) (24) as compared to the general population (10%) (25). As HD patients are more vulnerable to sarcopenia development, Ju et al. evaluated the preventive effects of leucine-enriched amino acid supplementation and resistance exercise in non-sarcopenic HD patients. After 12 weeks, the intervention induced significant improvement in muscle mass (increased in 64% of patients), strength (increased in 32% of patients), and physical function (improved in 60% of patients). This proposed intervention was a safe and effective way to prevent muscle deterioration in HD patients.

Patients under HD also suffer from a high prevalence of anemia (90–100%) (26), a common complication of CKD that can lead to adverse clinical outcomes (27). Erythropoiesis-stimulating agent (ESA) is the treatment of choice. However, as high doses of ESA increase the risk of all-cause mortality and cardiovascular (CV) events (27), strategies aimed to improve responsiveness need to be investigated. Lee et al. found an association between the ESA responsiveness and body composition in patients undergoing HD. Therefore, dietary and exercise interventions (such as the one from Ju et al. study) can have additional clinical benefits.

Obesity is an epidemic problem around the world, with increasing prevalence among CKD patients (28). Central obesity reflects visceral adipose tissue and is a well-known risk factor for CVD (29), the main cause of death in the CKD population (14). Diagnosis of central obesity is important not only to prevent CVD but also CKD progression as it can cause kidney damage (12). Among 35,018 participants from the National Health and Nutrition Examination Survey 2005–2018, Qin et al. found an association among visceral adiposity index and increased likelihood of decline in renal function (OR_adjusted_ = 1.04; 95% CI: 1.02–1.06) and albuminuria (OR_adjusted_ = 1.03; 95% CI:1.00–1.06). Visceral adiposity index was determined using waist circumference, BMI, TG, and high-density lipoprotein-cholesterol, parameters commonly available in clinical practice.

Among patients at stage 5 of CKD, Ryu et al. found that abdominal fat indices such as the conicity index and a-body shape index predicted CV outcomes and all-cause mortality and were associated with inflammatory status. Therefore, high central obesity is an important predictor of CV outcomes and may represent a mortality risk factor. Conicity index and a-body shape index are easily available in clinical settings, facilitating central obesity diagnosis.

In the last quarter of a century, there was an increased mortality and disability among patients with CKD. To prevent this undesirable scenario, there is a need for better understanding of the role of body composition in clinical and patient outcomes as well as the best intervention to prevent development of PEW, sarcopenia, and obesity. This volume brings a conjunction of articles aimed to fill this gap. We are certain that this reading will add new information to advance the research, promote knowledge transfer, and improve clinical practice in the field of CKD.

## Author contributions

NTB was involved in the Research Topic conception and drafted the editorial article. NTB, GB, and EPO critically revised articles submitted under the Research Topic. All authors critically revised the editorial manuscript.
